# The Simultaneous Effects of Spatial and Social Networks on Cholera Transmission

**DOI:** 10.1155/2011/604372

**Published:** 2011-12-07

**Authors:** Sophia Giebultowicz, Mohammad Ali, Mohammad Yunus, Michael Emch

**Affiliations:** ^1^Department of Geography, University of North Carolina at Chapel Hill, Saunders Hall, Campus Box 3220, Chapel Hill, NC 27599-3220, USA; ^2^Carolina Population Center, University of North Carolina at Chapel Hill, CB# 8120, University Square, 123 West Franklin Street, Chapel Hill, NC 27516-2524, USA; ^3^International Vaccine Institute, SNU Research Park, San 4-8, Nakseongdae-dong, Gwanak-gu, Seoul 151-919, Republic of Korea; ^4^International Center for Diarrheal Disease Research, GPO Box 128, Dhaka 1000, Bangladesh

## Abstract

This study uses social network and spatial analytical methods simultaneously to understand cholera transmission in rural Bangladesh. Both have been used separately to incorporate context into health studies, but using them together is a new and recent approach. Data include a spatially referenced longitudinal demographic database consisting of approximately 200,000 people and a database of all laboratory-confirmed cholera cases from 1983 to 2003. A complete kinship-based network linking households is created, and distance matrices are also constructed to model spatial relationships. A spatial error-social effects model tested for cholera clustering in socially linked households while accounting for spatial factors. Results show that there was social clustering in five out of twenty-one years while accounting for both known and unknown environmental variables. This suggests that environmental cholera transmission is significant and social networks also influence transmission, but not as consistently. Simultaneous spatial and social network analysis may improve understanding of disease transmission.

## 1. Introduction and Background

This study uses social network and spatial analytical methods simultaneously to understand cholera transmission in rural Bangladesh. Cholera is a bacterial disease that has been linked to the aquatic environment and can survive in brackish, coastal, and fresh water environments for significant periods of time [1–8]. Transmission occurs through the fecal-oral route, through two described forms [9–11]. Primary transmission is through direct contact with the pathogen and often occurs as seasonal events encourage growth of the bacteria in the environment. Secondary transmission occurs through person-to-person contact driven by human activity leading to contamination of shared food and water sources [12–16].

Network analysis is used to measure and explore relationships between people and to understand connections such as kinship, information sharing, or sexual contact [17–19]. Networks may also facilitate the spread of certain diseases or health outcomes. Much of the previous research in this field has focused on transmission of HIV/AIDS and STIs [20–24]. Tuberculosis, a respiratory illness that can spread through a single infected aerosol droplet, has also interested researchers due to its potential transmission through social interaction [19, 25, 26]. One previous study [27] used networks to study diarrheal disease risk at the community level, using spatial density of households and the volume of connections to other residences. Higher risk was due to crowding but lower risk was associated with social cohesion. Noninfectious health outcomes and health behaviors, such as obesity and smoking, are also examined through network analysis; these studies and others may integrate geographic components with social ties to measure any existing spatial effects [28, 29].

Health may be influenced by neighborhood-level environmental circumstances, as well as personal interactions through relationships. This study applies social network and spatial analytical methods simultaneously to model the transmission of cholera, which is a disease that may spread through social contact but also exists in environmental reservoirs. Indeed, cholera is shown to cluster in space, suggesting local-level risk factors [9, 30–32]. However, it may also spread through person-to-person contact. By using a combined social effects/spatial error model, spatial and social effects are detected while controlling for local environmental context. Such an integrated analysis can then provide evidence as to whether social behaviors and customs are significantly related to increased risk, especially in contrast to exposure through the environment.

## 2. Study Area and Data

**Figure 1:**
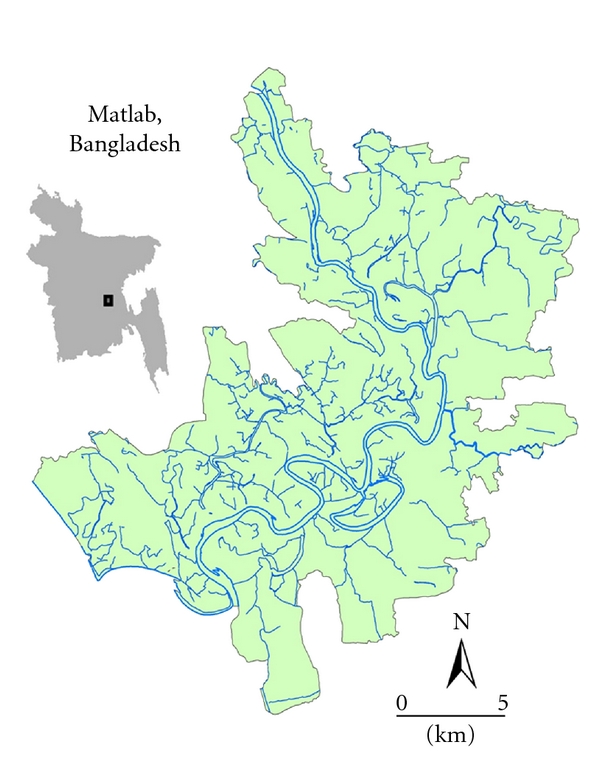
Matlab study area location within Bangladesh.

**Figure 2:**
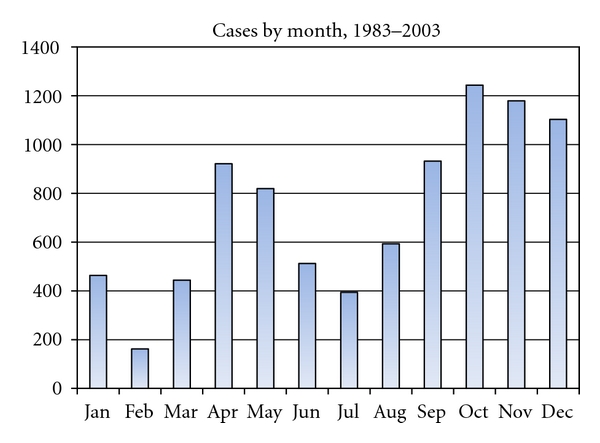
Total cholera cases in the population of Matlab by month, occurring between the study period of 1983 and 2003.

A combination of health, demographic, and geographic data is used to examine the social and spatial clustering of cholera in Matlab, the study area which is located in rural Bangladesh (Figure 1). The region receives over 75 inches of rain a year, much of which falls during the monsoon season between June and September. Poverty is high and diarrheal diseases remain endemic to the area. Cholera cases are typically most common during the pre- and post-monsoon period (Figure 2) [33].

Approximately 200,000 people reside in Matlab, clustered in *baris*, or groups of related households. *Baris *typically contain an average of five or six households [34]. The population of Matlab is monitored by the International Centre for Diarrhoeal Disease Research, Bangladesh (ICDDR,B) through a Health and Demographic Surveillance System (HDSS). Each resident, upon entry into the study area, through either birth or in-migration, is assigned a unique identification number within the database known as a Registration ID (RID). The individual is linked through this ID to a *bari* and household. Since a person can live in one *bari* initially but then relocate to another, every *bari* of residence for an individual is recorded in the HDSS, including dates of in- and out-migration. Community health workers visit each *bari* in Matlab once a month and record data on births, deaths, and migrations. Individuals who are sick are referred to and treated at the ICDDR,B hospital at no cost to the patient. Data on laboratory-diagnosed cases of diarrheal diseases are recorded at the hospital and then linked to demographic information for individuals, their *baris*, and households. Individual-level study data include the *bari* of residence of all Matlab residents between January 1st, 1983 and December 31st, 2003, dates of in- and out-migration, and all laboratory-confirmed cholera cases. A *bari*-level geographic information system (GIS) of Matlab was used to link cholera cases to locations.

## 3. Methods

The HDSS database was used to construct the kinship-based network. It contains the exact dates each person resided in different *baris*, therefore, a person can be traced from *bari* to *bari* over the course of his or her life in the Matlab study area. Migrations between *baris* are primarily kinship based, for example, due to marriage into a different family or to live near school or work. The actors in the network are thus individuals with some kinship relationship that leads to relocation from one physical residence to another. The kinship-based network was created and analyzed under the assumption that, when an individual moves, she/he maintains contact with the previous *bari* of residence at least for a short period of time. The mutual interaction between the old and new *baris* forms a nondirectional social connection. A limitation in using this type of network is that it does not encompass all social interactions in the lives of the Matlab population, only those that include family members. However, kinship-based relations are appropriate in this study population, given that these types of networks are an integral component of social interaction in lower socio-economic rural settings [35, 36]. Traditional customs such as shared meals and family visits encourage social and physical interaction between kin. Furthermore, by using the migration data, a comprehensive network is formed, because every individual in Matlab over the course of the study period is considered. Social connectivity data based on questionnaires and tracing contacts beyond the family would present a much smaller subsection of the entire network in Matlab.

Individual migrations thus form the connections between *baris*, which are the “nodes,” or units of analysis in the network. A migration from *bari x* to *bari y* creates a linkage between *bari x* and *bari y*, also known as a dyad. A list that contains all known connections between pairs in a network is called an edgelist. An edgelist can then be represented in a number of forms, such as a nodelist, a visual graph, or a matrix. A social adjacency matrix was used here, containing values of either 1 or 0, in which 1 represented the presence of a single, nondirectional social connection between two *baris* and 0 was the equivalent to no social connection. Multiple social networks and consequent matrices were created for each year of the study resulting in twenty-one individual networks.

Adding a spatial component involved creating a distance-based network to measure clustering, as well as creating environmental control variables. For each *bari*, all other *baris* located within a 1000-meter distance buffer were identified using the GIS. A distance-based spatial matrix of all *baris *was created, where 1 represented a common neighborhood between two *baris* and 0 represented no common neighborhood. The neighborhood buffer was then used to compare spatial clustering at various scales. For each *bari*, control variables were created based on proximity to environmental features that may be associated with cholera including rivers, ponds, tubewells, and roads. In addition, a variable for the depth of the nearest tubewell was included. The total number of *baris* used in the social and spatial network analysis was 8,873.

**Figure 3:**
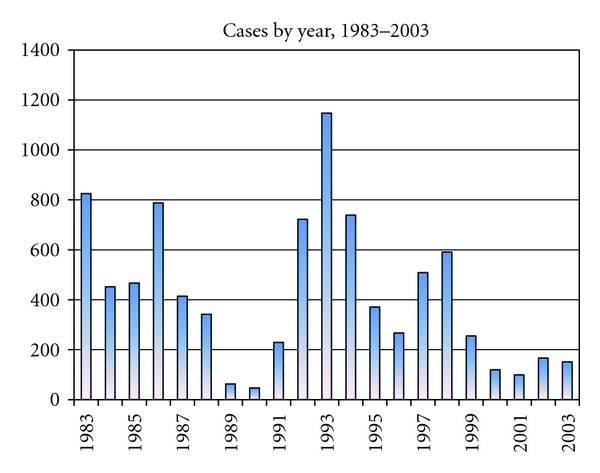
Total cholera cases in the population of Matlab by year.

The dependent variable for this study was the cholera incidence in a *bari* for each year. There were 8,765 laboratory-confirmed cholera cases in Matlab during the twenty-one-year study period (Figure 3). Cases were assigned to a *bari* for each year using the unique RID of the individual diagnosed with cholera at the ICDDR,B hospital. The total number of cases in that year was divided by the total population of the *bari*. Each year had an *n* × 1 vector of *bari*-level-dependent cholera values. The two 8,873 × 8,873 matrices, one for the social network (*W*_1_) and one representing the shared 1000-meter spatial neighborhood (*W*_2_), were row-standardized into weights matrices. Social affiliates and spatial neighbors were thus granted equal “weight” in terms of their influence on a particular *bari*. The matrices were then each multiplied by the *n* × 1 vector of cholera rates per *bari*, generating a lag operator which represents the average rate of cholera in neighboring *baris*, or those either socially affiliated (social lag) or spatially connected (spatial lag).

Identified social clustering may be due to spatial clustering, that is, individuals who are socially connected are more likely to live close to one another and be affected by the same environmental risk factors. Therefore, a combined linear spatial effects-spatial disturbance model estimating social effects while controlling for both known independent variables and unknown underlying spatial effects was built [37, 38]. The initial spatial effects model is represented as

(1) $$y=\rho_{1}W_{1}y+X\beta +e,$$

where *ρ*_1_ is the spatial effects parameter; *W*_1_ is the spatial weights matrix; *X* is a matrix of independent variable observations; *β* is a vector of parameters to be estimated; *e* is a randomly distributed error term. However, *e* could be spatially autoregressive, or the off-diagonal elements of the covariance matrix could also exhibit spatial dependence. In this case, the error term is represented as

(2) $$e=\rho_{2}W_{2}e+v,$$

where *ρ*_2_ is a spatial parameter for the disturbance term and *v* is an *n* × 1 vector of a randomly distributed error term. The model that integrates both spatial effects, or spatial lag, and spatial error appears as

(3) $$y=\rho_{1}W_{1}y+X\beta +\rho_{2}W_{2}e+v.$$

If estimated using typical OLS procedures it would be inefficient due to the autoregressive nature or correlation of the *Wy* term and the error term. Furthermore, the standard errors produced would be biased. Therefore, maximum-likelihood estimation methods are preferable for measuring the effects of interest [37].

The spatial effects-spatial disturbance model was implemented as described above, where *y* is the rate of cholera in a *bari* of interest. The primary difference is that *W*_1_ became a social weights matrix and *ρ*_1_ a social effects parameter. The remaining elements remained as described above, or the spatial weights matrix with a spatial disturbance term, and a random error component. The combined model was appropriate here, as the social effect, or the primary outcome of interest, was assessed in terms of both existence and strength, while the spatial disturbance was used to correct the bias potentially created by autocorrelation of cholera-related variables in space. Using the social and spatial weights matrices, the above model was run for each year using MATLAB 7.7.0 and the LeSage Econometrics Toolbox [39, 40].

## 4. Results

**Figure 4:**
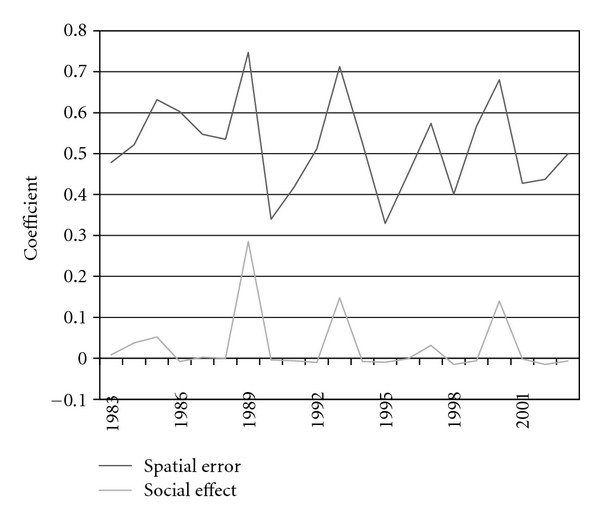
The coefficients representing the effect of social networks and spatial error, or unknown variables not captured in the model, on cholera incidence.

The results of the combined social effects/spatial error model are presented in Figure 4 and Table 1. Figure 4 graphically displays the coefficients for the spatial error and social effect of cholera clustering by year. Table 1 lists the coefficients, their significance levels, and the significant environmental control variables for each year. The spatial error was significant for every year at the *P* < 0.01 level. The parameter represents the extent to which the clustering of cholera rates, not explained by measured independent variables, nor the social effect, can be accounted for by the clustering of the error term. In other words, unaccounted-for variables related to similarity within the local environment were significant in all years. When this underlying spatial error was controlled for, the social effects parameter was significant at the *P* < 0.01 level for five out of twenty-one years, specifically for 1983, 1985, 1989, 1993, and 2000. The values represent the extent to which cholera rates clustered in the network; the lower coefficients are a result of the small number of overall cholera cases given the population size. The environmental control variables showed varying levels of significance in different years (Table 1).

## 5. Discussion and Conclusions

In a model identifying social clustering alone, visible concentration of cholera rates could be the product of socially connected *baris* located close to one another in space. Therefore, underlying spatial and environmental factors may be driving the similarity in cholera rates, as opposed to social network effects. The social effects-spatial disturbance model accounts for the spatial autocorrelation of omitted predictor variables or the autocorrelation of the error term. The model resulted in five years during which there was a significant social effect above and beyond spatial effects which were present in all years. During those five years, processes related to kinship-based social networks influenced cholera transmission and produced similar cholera rates in those *baris*. This may be the result of actual physical transmission of the pathogen through direct person-to-person transmission. It may also be due to similar behaviors across related *baris* that either increase or reduce collective risk. Examples would include hygiene or water storage practices.

Significant spatial error parameters estimated by the model for all years suggest the importance of unidentified spatial components in producing common cholera rates among socially connected *baris*. These components may include known risk factors identified in previous literature, such as population density or proximity to failing latrines. However, the spatial error parameter may “capture” those social interactions not included by the kinship network definitions. Individuals will interact to some degree with their neighbors. Whereas separating out these nonkinship networks is difficult without advanced survey methods, it is perhaps possible to use the available spatial information to predict social interaction. The Euclidian distance buffers used in this analysis represent the shorter distances that individuals commonly walk outside of their home. A limitation of this method is that it may ignore environmental features that obstruct access and therefore contact. Road and other transportation networks would be an optimal alternative to this, but these data are currently unavailable. However, larger Euclidian distance measures would most likely be highly correlated with transportation networks in Matlab. This analysis is only a first step to integrated spatial and social network analysis. For example, the choice of weights matrices that are created can affect findings, as parameter estimates are based on specification of either matrix *W* [41]. The social matrix in this study uses only a binary variable for either absence or presence of a social relationship. All relations that exist are given an equal value of 1 prior to standardization. Based on different theories of social influence, shared behavior, and interaction as related to social networks, a social weights matrix can be constructed in a variety of ways. Here a rather simplistic approach was used; alternatives would include weighting by number of connections (i.e., migrations) between two *baris* or by number of steps connecting the two *baris* (e.g., *baris* not directly related but sharing a connection with another *bari* would be given a value, such as 2). Furthermore, spatial distance, or spatial adjacency, can be represented in a variety of ways beyond the one-kilometer distance band. Varying distances may produce different results, as would using a weights matrix using absolute spatial distance between *baris*.

 Another consideration is temporal in nature, as using years as units may not capture effects related to cholera transmission at a seasonal or monthly scale. Matlab normally has two seasonal cholera epidemics per year during the pre- and post-monsoon periods. Previous studies have argued that different forms of transmission are responsible for the different epidemics; specifically, humans are more likely to drive the spread of cholera to other individuals during the pre-monsoon season, when water supplies are scarce [33, 42]. Using a finer time scale may reveal different patterns in the data. Additionally, the prediction of the dependent variable does not consider network ties and cholera cases from previous years, which may be significant. A longitudinal analysis would be appropriate and could be developed for many different time frames. Longitudinal social network analysis is only just beginning, and some studies have even begun to include a simple spatial component [29].

This project demonstrates not only that using social networks and accounting for spatial autocorrelation, social effects can be isolated as a cause of similar cholera incidence between *baris*, but that spatial and neighborhood-level factors are perhaps of greater importance due to their persistent effect over time. Development of targeted preventive intervention methods will be most effective if we understand the relative importance of primary and secondary transmission. In this study, we found that not only local neighborhood transmission is always important but also secondary transmission within kinship networks is also significant in some years.

## Figures and Tables

**Table 1:** Spatial error and significance, social effect and significance, and environmental variables that affect cholera rate.

Year	Spatial error	Significance	Social effect	Significance	Environmental control variables
1983	0.47	**	0.01	**	Pond**, tubewell*
1984	0.48	**	0.04		Road*
1985	0.58	**	0.05	**	Road*, pond*
1986	0.61	**	−0.01		Road**
1987	0.54	**	0.00		
1988	0.53	**	0.00		
1989	0.46	**	0.28	**	
1990	0.34	**	0.00		
1991	0.42	**	−0.01		
1992	0.52	**	−0.01		Pond*, tubewell*
1993	0.56	**	0.15	**	
1994	0.53	**	−0.01		
1995	0.34	**	−0.01		
1996	0.45	**	0.00		Tubewell depth**
1997	0.54	**	0.03		
1998	0.42	**	−0.01		River*, tubewell depth**
1999	0.57	**	−0.01		
2000	0.54	**	0.14	**	
2001	0.43	**	0.00		Road**
2002	0.45	**	−0.01		Tube*
2003	0.51	**	−0.01		

**P ≤ 0.01; *P ≤ 0.05.
